# Hippocampal sparing approach in fractionated stereotactic brain VMAT radio therapy: A retrospective feasibility analysis

**DOI:** 10.1002/acm2.12216

**Published:** 2017-11-10

**Authors:** Maria Daniela Falco, Stefano Giancaterino, Marco D'Andrea, Ramon Gimenez De Lorenzo, Marianna Trignani, Luciana Caravatta, Clelia Di Carlo, Saide Di Biase, Albina Allajbej, Domenico Genovesi

**Affiliations:** ^1^ Department of Radiation Oncology “G. D'Annunzio” University of Chieti SS. Annunziata Hospital Chieti Italy; ^2^ Laboratory of Medical Physics and Expert Systems National Cancer Center Institute Regina Elena Rome Italy

**Keywords:** fractioned stereotactic brain radiotherapy, hippocampal avoidance zone, hippocampal constraints, hippocampus sparing, VMAT

## Abstract

Volumetric Modulated Arc Therapy (VMAT) techniques for fractioned stereotactic brain radiotherapy (FSBRT) can achieve highly conformal dose distribution to intracranial lesions. However, they can potentially increase the dose to hippocampus (H) causing neurocognitive toxicity during the first four months after irradiation. The purpose of this study was to assess the feasibility of hippocampal‐sparing (HS) treatment plans in 22 patients with brain metastasis treated with VMAT technique. Firstly, we retrospectively analyzed hippocampal doses in all 22 VMAT original (not hippocampal‐sparing, NHS) plans. Plans with hippocampal dose exceeding constraints (9 out of 22) were re‐planned considering dose constraints on the hippocampus (H) and on hippocampal avoidance zone (HAZ) generated using 5 mm isotropic margin to the hippocampus. Conformity (CI) and homogeneity indexes (HI) on the target and MUs, were maintained as close as possible to the original plans. Mean CI_NHS_ and CI_HS_ obtained were: 0.79 ± 0.11 and 0.81 ± 0.10, respectively (*P* = 0.75); mean HI_NHS_ and HI_HS_ were 1.05 ± 0.02 and 1.04 ± 0.01 respectively (*P* = 0.72). In both sets of plans, the mean MU values were similar: 1033 ± 275 and 1022 ± 234 for NHS and HS respectively. In HS plans, the mean hippocampal dose was decreased by an average of 35%. After replanning, the D_max_ (21.3 Gy) for HAZ and H was met by 45% (4/9) and 78% (7/9) of the NHS plans, respectively. The worst results were obtained for cases with target volumes extention closer than 12 mm to H, because of the difficulty to spare hippocampus without compromising target coverage. After replanning D_40%_ constraint value (7.3 Gy) was met by all the 9 NHS plans. In conclusion, this study suggests that an hippocampal‐sparing approach to FSBRT is feasible resulting in a decrease in the dose to the hippocampus without any loss in conformity or increase in treatment time.

## 1 INTRODUCTION

Hippocampal injuries play a fundamental role both in short and long‐term memory loss and cognitive impairment.^1^, ^2^ Cranial irradiation can induce hippocampus damage, as suggested by some studies.^3^, ^4^, ^5^, ^6^, ^7^, ^8^, ^9^ In particular, cognitive impairment caused by whole brain irradiation (WBRT) has been investigated.^10^ These studies suggest that radiation‐induced neurocognitive toxicity occurs after irradiating neural stem cells of the hippocampus, potentially compromising patients quality of life (QoL).

WBRT has long been considered the mainstay treatment for patients with multiple brain metastases; nowadays, due to innovative technologies, fractioned stereotactic brain radiotherapy (FSBRT) and radiosurgery (SRS) can represent valid alternative therapeutic options to WBRT^11^, ^12^ allowing a better sparing of organs at risk, an improved outcome, and an increase in life expectancy; as a consequence, late onset radiation induced neurological sequels on hippocampi could be revealed in the course of life. Moreover, being the hippocampus very often close to the target, it could receive very high doses in extreme hypofractionated FSBRT treatments. Despite the large number of patients treated with these techniques, hippocampus is not routinely considered among organs at risk and the few clinical data available are not able to establish the correlation between dose on the hippocampus and cognitive effects. Results of the phase II RTOG 0933 study,^13^ show that some benefit in neuro‐cognitive functioning is achieved by hippocampal‐sparing in brain radiotherapy; however, phase III trial studies are necessary to validate the approach and confirm these findings. In spite of the paucity of clinical data, many authors focus on the feasibility of hippocampal‐sparing (HS) treatment plans. They applied the HS approach to WBRT followed by a radio‐surgical boost or WBRT and simultaneous integrated boost treatments using highly conformal techniques such as IMRT, helical tomotherapy or VMAT^14^, ^15^, ^16^, ^17^, ^18^, ^19^ demonstrating that HS plans were effectively able to spare the commonly delineated OARs including the hippocampus, while maintaining the same dose coverage and homogeneity of target volumes as the original ones. In these studies, the reduction in mean hippocampus dose was used as parameter to evaluate the appropriateness of the HS plans.

With regard to issues mentioned above, we conducted a retrospective feasibility study which consisted in elaborating HS plans for 22 FSBRT patients treated with VMAT technique maintaining the same coverage and homogeneity on the targets as the original plans. The hippocampus was firstly retrospectively delineated on the 22 plans and the corresponding dose–volume histograms (DVHs) were evaluated. Plans exceeding dose constraints for the hippocampus, were replanned introducing in the inverse planning module the dose volume constraints reported in literature.^7^, ^13^ The reduction in mean hippocampal dose in the new plans compared to the original plans, was evaluated.

## 2 MATERIALS AND METHODS

### 2.A Study design

The present study was a theoretical planning exercise aimed to test HS hippocampal sparing planning technique in FSBRT. We selected cases planned and treated using a VMAT technique without considering hippocampus as OAR; hippocampus was retrospectively delineated and hippocampus dose constraints were evaluated. To test HS VMAT feasibility, cases with hippocampus exceeding dose limits were re‐planned respecting original conformity and homogeneity indices.

### 2.B Patients and methods

A total of 22 cancer patients with 38 brain metastases were treated in FSBRT at the Radiotherapy Department, SS. Annunziata Hospital, University of Chieti. Sample and disease characteristics are reported in Table 1.

**Table 1:** Patients and tumor characteristics

Patients and disease characteristics	
Age (yr)	
Median	65
Range	48–84
Gender (n)	
Female	15
Male	7
Karnofsky performance status (n)	
70%	3
80%	5
90%	6
100%	8
Primary tumor (n)	
Lung	13
Breast	5
Colon‐rectum	4
Number of metastases (n)	
1	15
2	2
3	1
4	4
Metastatic site (n)	
Cerebellum	13
Parietal lobe	6
Frontal lobe	8
Other	11
Systemic disease control (n)	
Yes	16
No	6

All patients underwent a 2 mm thick non‐contrast computed tomography (CT) slice simulation and the images were acquired from the vertex to the lower border of C2. A thermoplastic mask with 3 fixation points was used as immobilization system. A 2 mm thick Magnetic Resonance Imaging (MRI) slice using a 3 Tesla MR scanner and gadolinium contrast‐enhanced T1 weighting was acquired for all patients. CT simulation and MRI images were co‐registered on Oncentra External Beam version 4.5.2 (Elekta Ltd., Crawley, UK) using a rigid registration algorithm. This coregistration was used to contour the targets (a single structure called CTV has been used as the composite of all the lesions), and organs at risk (OARs) such as optic chiasm and brainstem. The other OARs (lens, eyes, optic nerves, spinal cord, cochleae) were contoured using only CT data. Based on retrospective analysis of our clinical records, to account for setup and other treatment uncertainties, the planning target volume (PTV) was generated by adding a geometrical isotropic expansion of 3–5 mm to the CTV; a smaller isotropic expansion margin (3 mm) was applied in cases of optimal MRI‐simulation CT co‐registration. 11 patients received a total dose of 20 Gy (5 Gy/4 fractions) and 11 received 24 Gy (8 Gy/3 fractions). All VMAT plans were generated using the Collapse Cone Convolution algorithm in Plan Optimization Module (Oncentra Optimizer VMAT version 4.5.2) and delivered using Elekta Synergy Agility and 6 MV or 10 MV energies. The Agility multileaf collimator (Elekta AB, Stockholm, Sweden) had 160 leaves of projected width 0.5 cm at the isocenter. Maximum leaf speed was 3.5 cm/s.

To improve dose calculation accuracy for smaller lesions, the planning system was accurately extrapolated down to a 1 × 1 cm^2^ field size. The plans consisted in two full coplanar arcs and additional non‐coplanar partial arches were added to the two coplanar arcs with a couch angle chosen to avoid organs at risk. The dose normalization was chosen to ensure 95% of PTV volume coverage by 98% of prescribed dose (PD) for all of VMAT plans. In order to achieve better PTV coverage and lower OARs dose values, all VMAT plans were inversely planned optimizing leaf and gantry rotation speed and dose rate as free parameters. In case of irradiation of multiple lesions, the isocenter was automatically located at the center of mass of the lesions.

CBCT was performed daily before each treatment session to evaluate set‐up errors. CBCT acquisition volume (clip‐box) was determined to include whole PTV and OARs. The 3D‐3DCBCT‐ CT planning scan co‐registration was performed using the Gray level algorithm.

### 2.C Retrospective hippocampus study

The Hippocampus (H) was retrospectively delineated on the original plans by a radiation oncologist on gadolinium contrast‐enhanced T1‐weighted MRI. Delineation was performed on axial images using the RTOG 0933 atlas^20^ as reference. Afterwards, the contours in sagittal, coronal, and axial projections were revised by a neuroradiologist. A hippocampal avoidance zone (HAZ) was generated adding an isotropic 5 mm margin. The maximum dose (D_max_) and dose to 100% of hippocampus volume (D_100%_) were documented for all VMAT plans, according to the RTOG 0933 dosimetric compliance criteria.^13^ Moreover, the dose to 40% of hippocampus volume (D_40%_) was considered.^7^ Since RTOG hippocampal constraints were defined for a prescribed dose of 30 Gy in ten fractions, they were converted to biologically equivalent doses in 2 Gy fractions (EQD2). As the D_40%_constraint was more restrictive than D_100%_, the latter was no longer considered in this study.

Due to the alternative fractionation FSBRT schema, the analysis of DVHs was performed, for all the OARs, reconverting all dose values to equivalent doses in 2‐Gy fractions assuming an *α*/*β* ratio of 2 Gy.

In case original VMAT plans exceeded hippocampal constraints (non‐hippocampal‐sparing, NHS), HS plans were generated. The HS plans were elaborated following the same arc optimization systematic strategy as NHS regarding objectives, weights, and MU limit. Hippocampal constraints have been applied on HAZ. In addition, the distance between HAZ and the adjacent lesion was measured. The plan analysis included the evaluation of all the other OARs doses.

Dose delivery accuracy of all plans was assessed by measuring the 3D dose distributions with OCTAVIUS detector 729 device (PTW, Freiburg, Germany) and the agreement between measured and calculated dose profiles was checked using the gamma passing rate of 3% local dose (LD) and 3 mm distance to agreement (DTA) with a 10% threshold.

### 2.D Dosimetric evaluation of VMAT plans

Dosimetric evaluation of both NHS and HS plans was carried out by calculating conformity and homogeneity indexes. Healthy brain mean dose as a surrogate of the integral dose for said tissue^21^ was also evaluated.

The conformity index CI^22^ was defined as:

$$\mathrm{CI}=\frac{V_{T,pi}^{2}}{V_{T}\times V_{pi}}$$

where *V*_*T,pi*_ was the volume of the target covered by the prescription isodose, *V*_*T*_ was the target volume, and *V*_*pi*_ was the volume of the prescription isodose. The homogeneity index (HI),^23^ was computed as the ratio of the Maximum Dose (MD) to the PD.

$$\text{HI}=\frac{\mathrm{MD}}{\mathrm{PD}}$$

In presence of multiple PTVs, their cumulative volume was considered.

CI and HI values near to 1 correspond to more homogenous and conformal irradiation of the target volume. In the text, we refer to these indexes as CI_NHS_ and HI_NHS_ or CI_HS_ and HI_HS_ for NHS and HS plans respectively.

The plans were considered acceptable if the CI_HS_ and HI_HS_ values were found within ± 5% of CI_NHS_ and HI_NHS_ respectively. Maximum dose and doses to 40% of hippocampal volume (D_max_ and D_40%_, respectively) values were registered and compared to those of the corresponding NHS plans.

To evaluate healthy brain mean dose (NTMD), an additional structure [called non‐tumor (NT)] consisting of brain minus PTV was created. From DVHs, the mean doses were then extracted both for NHS (NTMD_NHS_) and HS (NTMD_HS_) plans.

## 3 RESULTS

Twenty‐two patients with 38 brain metastases were evaluated. Mean volumes for PTV and H were 5.7 cm^3^ (range 1–23.6) and 4.5 cm^3^ (range 2–6) respectively. The closest distance between the lesion and the hippocampal surface was found to be 2 mm (mean distance = 11.7 ± 6 mm). In NHS, the mean values of CI and HI were 0.79 ± 0.11 (range 0.54–0.93) and 1.05 ± 0.02 (range 1.02–1.08) respectively.

In 9/22 and 6/22 cases, D_max_ and D_40%_ constraints for HZA exceeded respectively. Constraints were not respected in patients with more than one metastatic lesion (six patients) and in three patients with only one lesion. For the nine plans exceeding D_max_ for HZA, hippocampal‐sparing plans were retrospectively elaborated.

For the new plans (HS plans), mean values of CI_HS_ and HI_HS_ were 0.81 ± 0.10 (range 0.59–0.92) and 1.04 ± 0.01 (range 1.03–1.07) respectively. These values were similar to CI_NHS_ and HI_NHS_ reported above although no statistical significance was found applying the *Pearson* chi‐square *test*, (*P* = 0.75).The lowest CI values, were observed for patients with more than one brain metastases.

HS plans maintained comparable MU values to NHS plans (mean MU values: 1033 ± 275 and 1022 ± 234 for NHS and HS respectively).

Table 2 reports CI and HI values for both NHS and HS plans (CI_NHS_ and HI_NHS_, CI_HS_ and HI_HS_), and all the distances from H to the closest lesion of nine patients with VMAT replanning. Table 3 reports NTMD values, both for NHS and HS plans (NTMD_NHS_ and NTMD_HS_) respectively. Mean NTMD_NHS_ value was found comparable with mean NTMD_HS_ (3.8 ± 1.2 Gy for NHS and 3.7 ± 0.9 Gy for HS plans, *P* = 0.73).

**Table 2:** CI and HI values for both NHS and HS plans of 9 patients with VMAT re‐planning. The last column reports the distance from H to closet target

# Patient	# Target	CI_NHS_	CI_HS_	HI_NHS_	HI_HP_	Distance from H to target (mm)
1	4	0.76	0.78	1.08	1.05	22
2	2	0.70	0.76	1.02	1.07	7
3	1	0.91	0.90	1.06	1.05	15
4	4	0.54	0.59	1.06	1.05	12
5	4	0.80	0.84	1.05	1.03	15
6	2	0.83	0.83	1.04	1.04	15
7	3	0.74	0.79	1.04	1.04	6
8	1	0.84	0.88	1.02	1.04	2
9	1	0.93	0.92	1.04	1.04	11

**Table 3:** NT mean doses (NTMD) for NHS and HS plans

# Patient	# Fractions	# Target	NTMD_NHS_ (Gy)	NTMD_HS_ (Gy)
1	5	4	4.3	4.4
2	5	2	4.2	3.9
3	5	1	1.8	1.8
4	5	4	4.0	3.8
5	5	4	4.7	4.5
6	8	2	3.5	3.6
7	8	3	5.3	4.9
8	8	1	3.0	2.9
9	8	1	3.4	3.6
Mean ± SD			3.8 ± 1.2	3.7 ± 0.9

Regarding constraints, passing from NHS to HS plans both for H and HZA, D_max_ decreased of 16% for H and 7% for HZA; a more pronounced variation was found for D_40%_ in HS plans with a decrease in about 40% both for H and HZA.

All D_max_ and D_40%_ values with dose values exceeding the constraints highlighted in gray, are recorded in Tables 4 and 5. As shown in Table 4, only 45% (4/9) of NSH plans with HAZ dose values exceeding D_max_ (max dose limit = 21.3 Gy) met the constraint after replanning. The number of HS plans respecting H constraints, increased slightly passing from 6/9 to 7/9 (about 78%). From the comparison of values reported in Tables 4 and 5 and those reported in Table 2, it can be deduced that the constraints were not respected in the case of the minimum hippocampus‐targets distance less than 12 mm. The closer hippocampus is to the target, the harder is to reduce the maximum dose without compromising target coverage.

**Table 4:** D_max_ values of 9 re‐planned cases. Dose values exceeding the constraints are highlighted in gray

# Patient	# Fractions	H		HAZ	
		D_max_(Gy)		D_max_(Gy)	
		NHS	HS	NHS	HS
1	5	12.9	10.5	21.8	15.2
2	5	29.9	15.7	34.1	33.9
3	5	10.5	7.9	24.5	17.0
4	5	20.9	21.0	28.7	28.2
5	5	20.4	15.3	24.0	21.0
6	8	15.5	12.8	23.8	20.0
7	8	39.8	36.7	54.6	55.6
8	8	58.9	59.0	60.5	61.1
9	8	17.0	11.7	27.1	26.4
Mean ± SD		25.1 ± 15.6	21.2 ± 16.5	33.2 ± 14.3	30.9 ± 16.6

**Table 5:** D_40%_ values of 9 replanned cases. Values exceeding the constraints are highlighted in gray

# Patient	# Fractions	H		HAZ	
		D_40%_(Gy)		D_40%_(Gy)	
		NHS	HS	NHS	HS
1	5	6.0	5.3	5.7	5.3
2	5	10.7	5.5	9.7	5.4
3	5	4.6	4.4	3.9	3.8
4	5	8.9	7.0	7.7	6.4
5	5	12.4	7.3	11.4	7.3
6	8	10.3	5.4	9.9	6.4
7	8	12.3	5.3	12.6	6.2
8	8	13.7	4.8	13.7	6.1
9	8	5.9	4.6	5.4	4.4
Mean ± SD		9.4 ± 3.3	5.5 ± 1.0	8.9 ± 3.4	5.7 ± 1.0

A different result was obtained for D_40%._ After re‐planning, D_40%_ constraint value (7.3 Gy) was met for all six NHS plans that originally did not comply for both H and HZA independent from the distance of the hippocampus from the target.

Three of the original plans had D_40%_ within the constraint for both H and HZA, but exceeded D_max_ constraint, so replanning was performed.

Finally, mean hippocampus doses decreased by an average of 35% in HS plans reaching values of 7.0 ± 3.4 Gy and 7.3 ± 3.2 Gy as compared to 9.9 ± 5.3 Gy and 9.8 ± 5.1 Gy in NHS plans for H and HAZ respectively.

Dose received by other evaluated OARs resulted below the constraints in both NHS and HS plans.

**Figure 1:**
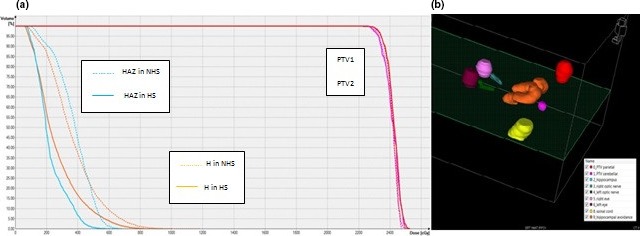
A cumulative normalized dose–volume histogram for hippocampus, hippocampal avoidance zone, and planning target volumes (PTV1 and PTV2) both in NHS and HS plans (Patient # 6 with two lesions) (a); the corresponding 3D structure view (b).

The DVH and 3D structure view of an HS plan for one representative patient (Patient # 6) is shown in Figs. 1(a) and 1(b), respectively. For this plan, both NHS and HS plan had the same CI (0.83) and H values (1.04). D_max_ and D_40%_ changed from 15.5 Gy to 12.8 Gy and 10.3 Gy to 5.4 Gy, respectively, for H and from 23.8 Gy to 20.0 Gy and 9.9 Gy to 6.4 Gy, respectively, for HZA.

**Figure 2:**
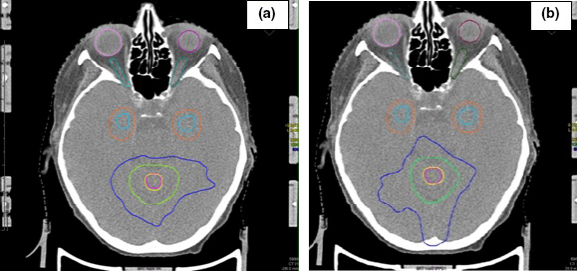
Spatial dose distributions in axial view for a representative NHS (a) and HS (b) VMAT plans (Patient # 6). In the box of Fig. 1 the contoured regions are listed. Blue isodose represents 6 Gy (D25%);green, 12 Gy (D50%) and yellow 22.8 Gy (D95%). Total dose of 24 Gy, in three fractions.

**Figure 3:**
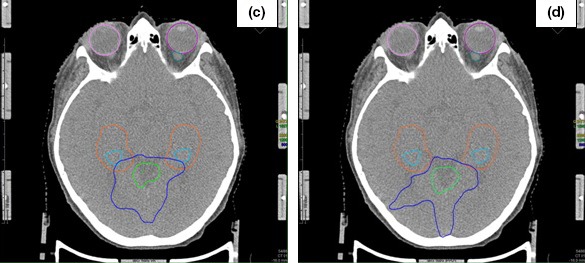
Spatial dose distributions in axial view for the same representative patient but taken in a different slice. In the box of Fig. 1 the contoured regions are listed. Blue isodose represents 6 Gy (D25%);green, 12 Gy (D50%), and yellow 22.8 Gy (D95%). Total dose of 24 Gy, in three fractions.

Figures 2 and 3 show two different axial sections (z = 26 mm and z = 16 mm, respectively) of the corresponding dose distribution. Figure 2 represents the axial view referred to (a) NHS; (b) HS plan. By comparing (a) and (b), no significant difference in dose distribution seems to exist. The situation changes if we consider the axial section corresponding to z = 16 mm, shown in Fig. 3. In (c) (NHS plan), the 6 Gy isodose is inside HZA structure and touches H; in (d) it slightly touches HAZ (HS plan) only, with a greater dose sparing for both HZA and H.

All treatment plans presented gamma passing rate of at least 91% (LD = 3%, DTA = 3 mm).

## 4 DISCUSSION

The role of the hippocampus in memory function is well‐established in literature^1^, ^2^; although some studies affirm that cranial irradiation may damage neurogenic stem cells located in the subgranular layer of the hippocampal dentate gyrus inducing neurocognitive toxicity,^3^, ^4^, ^5^, ^24^, ^25^ dose constraints for hippocampus have not yet been elucidated, making it difficult to establish the potential benefits of hippocampal‐sparing approach. Some constraints were derived from the initial results of an analysis of patients affected by brain metastasis undergoing WBRT (D_max_ < 16 Gy and D_100%_ < 9 Gy at 3 Gy per fraction for a total dose of 30 Gy) and low/high grade gliomas receiving fractionated stereotactic radiation therapy (D_40%_ < 7.3 Gy on bilateral hippocampus at 2 Gy per fraction).^7^, ^10^, ^13^

D_max_ and D_100%_ constraints were also adopted by Pokhrel et al.^26^ who retrospectively investigated the plan quality and accuracy of using hippocampal‐sparing intensity modulated arc therapy in WBRT treatments.

In another works, the mean dose was considered as dose reference to optimize plans in a hippocampal‐sparing approach when highly conformal techniques such as IMRT, helical tomotherapy or VMAT are used.^14^, ^15^, ^16^, ^17^ Gondi et al^14^ compared HS plans with standard WBRT ones where a homogenous dose 30 Gy was applied to the whole brain including hippocampus. HS plans aimed to decrease the mean hippocampal dose obtaining 5.5 Gy (D_max_ 12.8 Gy) and 7.8 Gy (D_max_ 15.3 Gy) values for helical tomotherapy and LINAC based RT respectively. Gutierrez et al^15^ and Hsu et al^16^ focused on elaborating plans with mean dose in WBRT simultaneous integrated boost treatments followed by radio‐surgical boost or WBRT using tomotherapy^15^ and VMAT^16^ techniques respectively. The mean dose was also considered as a parameter that should be considered for developing hippocampal‐sparing strategies in patients undergoing WBRT followed by radio‐surgical boost^18^ or WBRT and simultaneous integrated boost^19^ treated with VMAT. Hippocampal avoidance strategies have been also implemented for locally advanced nasopharyngeal carcinoma^9^ and in radiosurgery of multiple intracranial targets using Gamma Knife Perfection^®^ equipment.^27^ Both studies confirm the reduction in mean hippocampal dose up to 35% in hippocampal‐sparing replanned cases, maintaining the same conformity and target coverage. Furthermore, it should be clarified if hippocampus dose constraints should be applied only to hippocampus or else to hippocampal avoidance zone. However, some authors indirectly suggest the “non‐applicability” of constraints to HAZ observing that sparing HAZ (hippocampus + 5 mm margin) poses a theoretical risk of disease progression in the margin region.^6^

Taking into account these issues, we evaluated the feasibility of using VMAT to deliver FSBRT with hippocampal avoidance. To our knowledge, similar data do not exist in literature, either considering H or HZA. In this study, we replanned 9 of 22 FSRT VMAT plans (HS plans) since they had both HA and HAZ exceeding the considered EQD2 limits which were: D_max_ < 21.3 Gy and D_40%_ < 7.3 Gy, respectively.^7^, ^10^, ^13^ HS VMAT plans, maintained the same conformity (*P* = 0.75) and homogeneity (*P* = 0.72) of the original plans (NHS). The mean CI_NHS_ and CI_HS_ values were: 0.79 ± 0.11 and 0.81 ± 0.10 respectively. These were in good agreement with data reported in literature, for multiple no coplanar arcs in VMAT techniques in presence of single or multiple cranial lesions and single isocenter^28^, ^29^, ^30^. In particular, we found the same dependence of the average CI values on the number of lesions. Infact, our values spread from 0.59 in case of 4 lesions to 0.92 in case of 1 lesion and HS. In literature, an average value of 0.86 is reported in the case of two lesions^29^ and an lower average value of 0.63^30^ in case of multiple lesions. Regarding HI, Wang et al.^30^ reported a mean value of 1.15 ± 0.03 higher than our (1.05 ± 0.02 in the NHS plans and 1.04 ± 0.01 in the HS plans); the higher number of lesions treated by Wang, however, could have limited the homogeneity of the plans. Our results show that plan quality after hippocampal sparing is still well within the published standards of conformity and homogeneity. Mean NTMD_NHS_ value was found comparable with mean NTMD_HS_ (*P* = 0.73). This means that changing treatment parameters passing from NHS to HS plans maintaining the same target coverage does not increase the total energy deposited to the healthy brain.

In our retrospective analysis, we found that 9 of 22 cases had D_max_ above the limits; 45% of them were recovered for HAZ (4/9 plans); one more plan was recovered for H (they passed from 6/9 to 7/9 plans!); dose values at 40% of volume (D_40%_) in HS plans were all recovered, both for H and HAZ. For HS plans presenting target adjacent to H (less than 12 mm) D_max_ was not recovered, regardless the number of the lesions and dose prescription.

Even though mean doses in HS plans were not considered as constraints in the optimization strategy, the obtained values were in good agreement with the values reported in literature and close to the constraint proposed by several authors (6 Gy).^14^, ^15^, ^16^, ^18^ However, it is difficult to compare our data with literature evidence, because dose prescription and fraction number are different, compared to similar studies regarding RT treatments for patients affected by metastatic brains but treated with different techniques, prescription doses and volumes (mainly WBRT followed by a sequential boost, or WBRT with concomitant boost). In any case, we noticed a reduction in about 35% in the mean dose and D_40%_ of hippocampal volume in HS plan in comparison to NHS plans suggesting that these two parameters are more sensible than D_max_ to optimization procedures.

In summary, our data suggest that for FSBRT performed with VMAT technique (a) targets adjacent to hippocampus could only partially benefit from an hippocampal‐sparing approach, in case good conformity and target coverage is needed considering D_max_ as a rigid constraint in the optimization tool; and (b) the doses undergoing a more important decrease (mean dose and D_40%_), may benefit much more from HS approach. On the other hand, our HS plans were optimized with respect to D_max_ and D_40%_. In a future work, we intend to investigate the impact of D_40%_ and the mean dose as constraints in the optimization tool in HS approach and use the constraint on D_max_ only in selected cases. In any case, the total number of MU did not change significantly among NHS and HS plans, so we think that an hippocampal‐sparing approach should always be attempted.

## 5 CONCLUSION

This study suggests that hippocampal‐sparing approach in fractionated stereotactic brain radiotherapy VMAT treatments is feasible, resulting in an overall decreased dose to the hippocampus. HS plans maintain the same target conformity and homogeneity, the same mean dose to surrounding healthy tissues and the same treatment time of the original plans. In case of hippocampal distance from the target larger than 12 mm, all the considered dose constraints are respected. Anyhow, a reduction in 35% has been obtained for the mean dose and D_40%_. Although safe threshold doses for the hippocampus have not been defined yet, it is strongly advisable to delineate the hippocampus and put in practice all the necessary strategies to reduce doses especially in patients with a reasonable life expectancy.

## CONFLICT OF INTEREST

The authors declare that they have no conflict of interest.
